# Regulation of the mitochondrial reactive oxygen species: Strategies to control mesenchymal stem cell fates ex vivo and in vivo

**DOI:** 10.1111/jcmm.13835

**Published:** 2018-08-30

**Authors:** Chenxia Hu, Lingfei Zhao, Conggao Peng, Lanjuan Li

**Affiliations:** ^1^ Collaborative Innovation Center for Diagnosis and Treatment of Infectious Diseases State Key Laboratory for Diagnosis and Treatment of Infectious Diseases School of Medicine First Affiliated Hospital Zhejiang University Hangzhou Zhejiang China; ^2^ Kidney Disease Center The First Affiliated Hospital College of Medicine Zhejiang University Hangzhou Zhejiang China; ^3^ Key Laboratory of Kidney Disease Prevention and Control Technology Zhejiang Province, Institute of Nephrology Zhejiang University Hangzhou Zhejiang China

**Keywords:** mesenchymal stem cell, multilineage, reactive oxygen species, regenerative medicine, self‐renewal

## Abstract

Mesenchymal stem cells (MSCs) are broadly used in cell‐based regenerative medicine because of their self‐renewal and multilineage potencies in vitro and in vivo. To ensure sufficient amounts of MSCs for therapeutic purposes, cells are generally cultured in vitro for long‐term expansion or specific terminal differentiation until cell transplantation. Although physiologically up‐regulated reactive oxygen species (ROS) production is essential for maintenance of stem cell activities, abnormally high levels of ROS can harm MSCs both in vitro and in vivo. Overall, additional elucidation of the mechanisms by which physiological and pathological ROS are generated is necessary to better direct MSC fates and improve their therapeutic effects by controlling external ROS levels. In this review, we focus on the currently revealed ROS generation mechanisms and the regulatory routes for controlling their rates of proliferation, survival, senescence, apoptosis, and differentiation. A promising strategy in future regenerative medicine involves regulating ROS generation via various means to augment the therapeutic efficacy of MSCs, thus improving the prognosis of patients with terminal diseases.

## 1 INTRODUCTION

Mesenchymal stem cells (MSCs) are broadly used in cell‐based regenerative medicine because of their self‐renewal and multilineage potencies in vitro and in vivo. These cells can be easily isolated from various tissues and then cultured over multiple passages for further application.^1^ After transplantation in vivo, they migrate and repair injured tissues or organs, thus making a contribution to tissue engineering; the fracture site is able to induce senescence or apoptosis based on the surrounding harsh conditions and oxidative stress. However, MSCs gradually lose their proliferation and differentiation potential after long‐term ex vivo culture. To ensure sufficient amounts of active MSCs for cell therapy, the surrounding microenvironment is considerably important for MSCs in vitro and in vivo.

**Figure 1:**
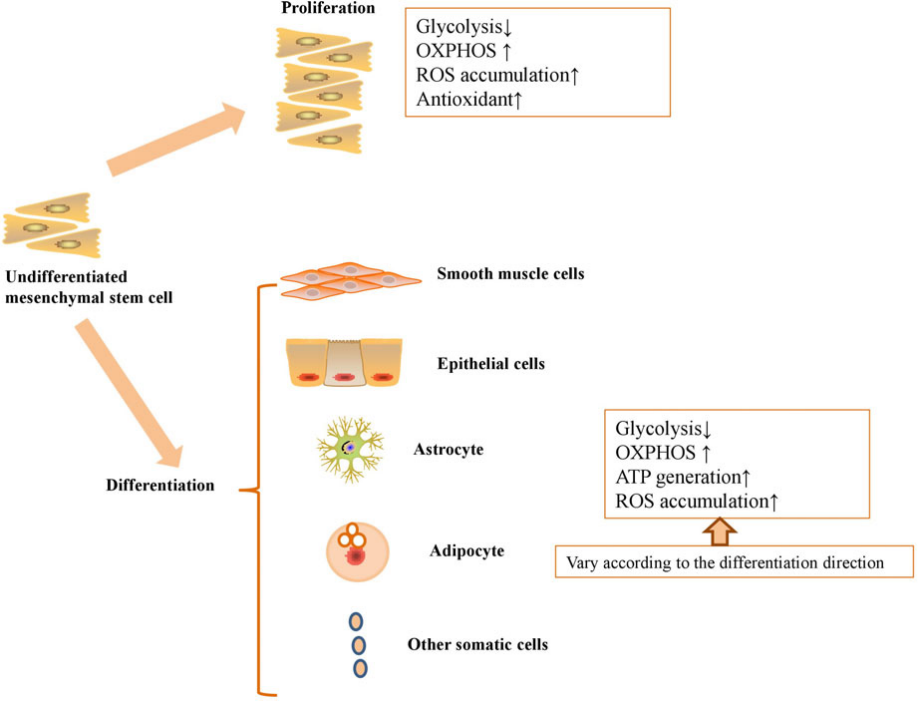
Physiologically up‐regulated ROS production is essential for MSC proliferation and differentiation

Under physiological conditions, adenosine triphosphate (ATP) production can be generated through glycolysis and oxidative phosphorylation (OXPHOS), while mitochondrial respiration generate a certain amount of reactive oxygen species (ROS) as byproducts. NADH‐coenzyme Q oxidoreductase (Complex I) and ubiquinol‐cytochrome c oxidoreductase (Complex III) release electrons for generation of superoxide anion (O_2_‐.).^2^ Superoxide dismutase (SOD) will be activated for generation of hydrogen peroxide (H_2_O_2_) and then H_2_O_2_ will be decomposed to O_2_ and H_2_O after activation of catalase (CAT) or glutathione peroxidase (GPx).^2^ In addition, other metabolic intermediates including 2‐oxoglutarate dehydrogenase, pyruvate dehydrogenase, glycerol 3‐phosphate dehydrogenase also contribute to the upregulated generation of ROS in MSCs.^3^ ROS has been commonly thought of as harmful to cell functioning and can lead to apoptosis and senescence of MSCs in vitro. For example, Geissler et al^4^ demonstrated that MSCs upregulated the level of ROS after long term culture for several passages, accompanied with the impaired mitochondrial function and differentiation ability. In addition, the upregulated ROS can cause lipid peroxidation, oxidative modification of proteins, DNA damage and cellular senescence.^5^, ^6^ However, physiologically up‐regulated ROS production is essential for stem cell activities. In an undifferentiated state, MSCs have low levels of intracellular ROS and high levels of antioxidative enzymes^7^; while differentiated MSCs show the opposite properties.^8^ On the other hand, physiologically up‐regulated ROS are required for MSC proliferation, while inhibition of ROS blocks MSC self‐renewal^9^ (Figure 1).

Commonly, ROS can be generated by mitochondria,^10^ endoplasmic reticulum (ER),^11^ cytosol,^12^ peroxisomes,^13^ plasma membrane,^14^ and extracellular space.^15^ Aerobic microenvironment brings out reduction of molecular oxygen, and then Ero1p will consequently yield stoichiometric H_2_O_2_.^11^ All‐trans arachidonic acid is able to improve the generation of ROS via xanthine dehydrogenase/xanthine oxidase interconversion in the in vitro liver cytosol.^12^ After peroxisomes are supplemented with uric acid, H_2_O_2_ will be generated at a rate which corresponds to 42%‐61% of the rate of uric acid oxidation.^13^ Fibroblast contains an ectoplasmic enzyme, distinct from NADPH oxidase, which can generate a major source of ROS after tissue damage.^14^ NADPH oxidase maintains endothelial cell xanthine oxidase activity for upregulating ROS generation in response to oscillatory shear stress.^15^ Although ROS are generated by various organelles, the mitochondrion is the main organelle of ATP and ROS generation.^16^ Respiratory chain electrons escape the mitochondrion, leading to superoxide ion and H_2_O_2_ generation due to superoxide dismutation via SOD catalysis in MSCs.^17^, ^18^, ^19^, ^20^ Overproduction of ROS enhances autophagy and apoptosis in MSCs through activation of c‐Jun NH(2)‐terminal kinase (JNK) signalling.^21^ In this process, colocalization of ataxia telangiectasia mutated (ATM), histone H2A.X, and p53‐binding protein 1 (53BP1) results in Bcl‐2‐associated X protein (BAX)‐Bcl‐2 homologous antagonist/killer (BAK) dimerization; subsequent release of cytochrome c to the cytoplasm and caspase release triggers apoptosis.^22^, ^23^ For cellular homeostasis, endogenous scavengers help to remove excessive ROS, including the following: enzymatic proteins such as SOD, peroxiredoxins, glutathione peroxidase and lysosomal catalases; and non‐enzymatic antioxidants such as vitamins, carotenoids and flavonoids.^24^, ^25^, ^26^, ^27^ Under physiological conditions, mitochondrially derived ROS include superoxide anion, hydroxyl radical, singlet oxygen, hydrogen peroxide, nitric oxide, and peroxynitrite.^28^ However, the incomplete oxidation of oxygen to water by mitochondrial complexes leads to excessive ROS production. Various conditions including ageing, long‐term culture and H_2_O_2_, adverse oxygen content, high glucose, proinflammatory cytokines and other toxic factors, abnormally up‐regulated ROS production can harm MSC activities.^22^, ^29^, ^30^, ^31^, ^32^, ^33^ On the other hand, although MSCs play critical roles on immunomodulatory properties for therapy in various diseases, the constant isolation from donors also bring out unrecoverable injury.^34^

In the current review, we mainly discuss the known ROS generation mechanisms and the regulatory routes for controlling MSC fates. Some key signalling pathways for maintaining energy metabolism and ROS homeostasis play particularly important roles in regulating MSC fates ex vivo and in vivo (Table 1). Based on these regulatory pathways, it may be possible to control MSC fates in vitro and in vivo by regulating ROS levels surrounding MSCs for future regenerative medicine.

**Table 1:** The key signalling pathways for maintaining energy metabolism and ROS homeostasis of MSCs ex vivo and in vivo

Factor	Signalling pathway/mechanism	Effect on MSCs	Ref
Age	DNA methylation status↑	Oxidative stress↑; mitochondrial function↓	29
Long‐term culture	ROS‐induced suppression of c‐Maf↑	Proliferative ability↓; differentiation capacity↓	30
Long‐term culture	p38↑; MAPK↑; p53/p21↑	Senescence↑	70
H_2_O_2_	p53↑; p21 ↑; p38↑; pRb↓	Cell cycle↓; DNA damage↑	22
Adipogenic differentiation	mTOR↑; NOX‐4↑; FOXO↑	PPARγ↑	55, 56, 57, 58, 59
Chondrogenic differentiation	AKT↑; ERK↑	NOX‐2↑; NOX‐4↑	8
Osteogenic differentiation	Wnt/*β*‐catenin↑	ROS↑; apoptosis and necrosis rates↑	60, 61
*β*‐ME	Notch1↓	ROS↓; neural stem cell‐specific proteins↑	62
Particulate matter	AKT↓	MSC proliferation↓; ROS↑	36
Old rat serum	Wnt/*β*‐catenin↑	MSC proliferation↓; ROS↑	37
FGF‐23	p53↑; p21↑	Senescence↑	33
TNF‐α	NF‐κB↑	Survival rate↑; migratory capacity↑	39
Atmospheric oxygen	p53↑	Viability↓; cell growth↓	31
Hypoxia	AKT↑; mTOR↑	Proliferative capacity↑; differentiation↑	43, 44
Interleukin‐17	MEK‐ERK↑	MSC proliferation↑; migration↑; motility↑	83
PEDF	p53↓; p16 ↓	Cell survival rates in long‐term culture↑	85
NAC	Wnt/*β*‐catenin↓	Self‐renewal↑, multipotency↑; apoptosis rate↓; cell adhesion capacity↑	60, 89, 90, 91
Fucoidan and carvedilol	p38‐MAPK↓; JNK↓; caspase 3↓; AKT↓	H_2_O_2_‐induced injury↓	101, 102
AGEs	ROS‐p38 mediated pathway↑	Proliferative capacity↓; migratory capacity↓	49
Apocynin	NOX↓	Ageing process↓; osteogenesis↑ of ageing MSCs	108
Lycopene/Cirsium setidens	p38‐MAPK↓; JNK↓; ATM ↓; p53↓	H_2_O_2_‐induced ROS generation↓; survival rate↑	109, 110
CoQ10	AKT/mTOR↑	D‐galactose‐induced MSC ageing↓; ROS↓	137
Menadione and 2,3‐dimethoxy‐1,4‐naphthoquinone	ERK1/2↑; JNK1/2↑	Migration capacity↑	116
Cholesterol	ROS/p53/p21Cip1/Waf1 signalling pathway ↑	MSC senescence↑	117
High‐density lipoprotein	PI3K/Akt pathway↑	Cell viability↑; apoptosis↓	118
miR‐210 inducers	ERK1/2↑; AKT ↑; c‐Met↓	ROS‐induced apoptosis rate↓	82, 132
Knockout of exon 4 of pNO40/PS1D in MSCs	p16↑; Rb ↑	Ageing↑; osteogenic differentiation defect↑	124

## 2 SURROUNDING MICROENVIRONMENTS FOR REGULATING ROS PRODUCTION IN MSCS

Stem cells have been assumed to rely on anaerobic energy metabolism, as they are always in a hypoxic microenvironment before they are isolated from the source tissues. Because MSCs cultured in vitro are sensitive to the surrounding microenvironment, optimization of culture conditions is important for long‐term culture without loss of stem cell properties.

Contact culture at 100% confluence significantly increases ROS levels and promotes MSC senescence without influencing expression levels of telomerase reverse transcriptase (TERT) and p53.^35^ Moreover, when added to the culture medium, multiple factors have a negative impact on MSC properties. For example, particulate matter or old rat serum inhibits MSC proliferation and increases ROS formation by attenuating signalling via AKT phosphorylation and activating Wnt/β‐catenin signalling, respectively.^36^, ^37^ On the other hand, fibroblast growth factor 23 (FGF‐23) promotes MSC senescence by up‐regulating p53 and p21 expression levels,^33^ and TGF‐β1 significantly down‐regulates SOD2 and Id1 expression levels in MSCs, thus increasing levels of senescence‐related genes in a dose‐dependent manner.^38^ In contrast, preconditioning MSCs with tumor necrosis factor alpha (TNF‐α) strongly enhances their proliferative and migratory capacities; indeed, this cytokine effectively increases the survival rate and migratory capacity of MSCs even under oxidative stress in vivo.^39^ Furthermore, high glucose increases the expression levels of pluripotent markers in MSCs but does not alter the adipogenic and osteogenic differentiation capacities of the cells; it also decreases proliferation and migratory capacities of MSCs by enhancing ROS levels.^32^ Hypoxic conditions can decrease the expression levels of antioxidants, including glutathione, glutathione peroxidase and SOD1, and can increase the apoptosis rates of MSCs.^40^ Under culture conditions of extreme hypoxia and exposure to metal carcinogens, MSCs exhibit increased levels of oncogenic proteins and decreased levels of tumour suppressor proteins yet aberrant proliferation rates and redox mechanisms via intracellular ROS accumulation in vitro.^41^ In contrast, hypoxic conditions may also protect MSCs from injury, promoting proliferation, differentiation, and survival in a ROS‐mediated manner. A paradoxical discovery was that compared with normoxia, MSCs cultured under hypoxia showed a smaller size and greater cellular complexity, lower proliferation and mitochondrial activity, and reduced autophagy.^42^ It also moderately increases ROS production and promotes the proliferative capacity and differentiation potency of MSCs via phosphorylation of the AKT and mTOR pathways.^43^, ^44^ Intriguingly, long‐term hypoxic conditions upregulated the expression of HIF‐1α, and the hypoxia MSCs showed greater cell viability, decreased ROS levels and increased resistance to oxidative stress for alleviating both early radiation‐induced pneumonia and late pulmonary fibrosis when compared to normoxia MSCs.^45^ In addition, a low dose of hypoxic MSCs but not a high dose of hypoxic MSCs can effectively attenuate ischemia/reperfusion (I/R) induced permeability pulmonary oedema by decreasing ROS related inflammatory responses and anti‐apoptosis effect.^46^ On the other hand, Karlsen et al^47^ showed that hyperoxia of room air was not adverse for the proliferation, differentiation, or phenotype of bone marrow derived MSCs in vitro. Saini et al^48^ demonstrated that preconditioned with hyperoxia decreased the apoptosis of MSCs, while increased the the level of survival markers including Akt1, NF‐κB, and Bcl‐2. However, whether hyperoxia will alter the MSC activities via ROS related pathway need to to be further investigated. Advanced glycation products (AGEs) can dose‐dependently decrease the proliferative and migratory capacities of MSCs via activation of the ROS‐p38 mediated pathway in a dose‐dependent manner.^49^ In fact, addition of AGEs significantly enhances the levels of osteogenic markers while inhibiting the maturation of osteogenic MSCs; moreover, AGEs inhibit MSC adipogenic and chondrogenic differentiation.^50^

## 3 ROS GENERATION AND THE IN VITRO DIFFERENTIATION FATE OF MSCS

In general, undifferentiated MSCs have fewer mitochondria than differentiated MSCs, though the mitochondrial copy number, OXPHOS supercomplex, SOD expression, mitochondrial biogenesis and ROS levels are all significantly increased after specific differentiation.^51^, ^52^ ROS imbalance will lead to a significantly reduced MSC differentiation capacity,^53^ thus limiting MSC applications in vitro and in vivo. When adult MSCs are incubated under stress‐inducing conditions, they are considered to be in a state of cyclic stretch and produce more ROS and have weaker differentiation capacities.^54^ However, stimulation by different factors will induce MSCs to differentiate into various terminal somatic cells; according to current studies, different differentiation directions lead to various degrees of ROS production. The mammalian target of rapamycin (mTOR),^55^ mitochondrial biogenesis,^56^ NOX‐4,^57^ and forkhead box O (FOXO)^58^ pathways are activated after adipogenic differentiation of MSCs, and ROS expression levels are strongly enhanced. This excessive ROS causes a positive feedback on activating peroxisome proliferator‐activated receptor gamma (PPARγ) to accelerate adipogenic differentiation.^59^ Similarly, chondrogenic differentiation of MSCs has been demonstrated to activate AKT and extracellular signal‐regulated kinase (ERK) signalling and to up‐regulate expression of ROS‐related proteins, including NOX‐2 and NOX‐4. Excessive levels of ROS also lead to positive feedback on the chondrogenic progress.^8^ Osteogenic differentiation in MSCs significantly activates the Wnt/β‐catenin pathway and increases intracellular ROS levels, and the chromatin fragmentation caspase 3 expression induced by osteogenic differentiation consequently results in increased apoptosis and necrosis rates in MSCs.^60^, ^61^ As a type of antioxidant, β‐mercaptoethanol (β‐ME) is able to gradually increase the expression levels of neural stem cell‐specific proteins via down‐regulation of Notch1 expression and ROS production.^62^ However, Esmaeli et al^63^ demonstrated that although umbilical cord derived MSCs decreased the levels of polyunsaturated fatty acids and gradually enhanced the levels of saturated fatty acids after hepatogenic differentiation, these effects were independent of ROS production and lipid peroxidation at the end stage of hepatic differentiation. Therefore, the relationship between ROS generation and MSC differentiation fate should be elucidated before any conclusions regarding how to direct ROS towards controlling the direction of differentiation can be drawn.

## 4 ANTIOXIDATIVE CAPACITIES OF SENESCENT AND APOPTOTIC MSCS

Age, long‐term culture and H_2_O_2_ are important factors for inducing MSC senescence and can easily alter mitochondrial morphology, decrease antioxidant capacities, elevate ROS levels and increase the apoptosis rate. Reduced proliferation and differentiation capacities consequently decrease the therapeutic potential of MSC‐based treatments.^4^, ^64^, ^65^ Aged MSCs undergo senescence through increased DNA methylation, oxidative stress and mitochondrial function impairment.^29^ However, controversy remains regarding the pluripotency as well as the migration and antioxidative capacities of aged MSCs, as Lund et al^66^ demonstrated that all of the above features are equivalent in bone marrow derived MSCs from young and old individuals. Another studies conflict with the assertion that donor age negatively impacts MSC suppression of T cell proliferation^67^, ^68^; one of these studies analysed 53 human donors ranging within 13‐80 years demonstrated no significant correlation between age and T cell suppression capability.^67^ Moreover, although Li et al^69^ demonstrated that MSCs derived from old donors showed a more rapidly decreased survival rate than MSCs derived from young donors in the infarct region in acute myocardial infarction (MI) model, N‐acetyl‐L‐cysteine (NAC) which is a ROS scavenger can protected MSCs derived from old donors from apoptosis in vivo and significantly enhanced the therapeutic effects of MSCs. Long‐term culture always results in proliferative decline, cell cycle arrest and decreased differentiation capacity in adipose‐derived MSCs by ROS‐induced suppression of c‐Maf.^30^ However, long‐term culture of umbilical cord blood‐derived MSCs reinforces senescence by activating p38, MAPK, and p53/p21 pathways and enhancing ROS generation.^70^ According to the basic classification, long‐term cultured MSCs can be divided into early passaged MSCs and late‐passaged MSCs. With increasing passage number, ROS production and the apoptosis rate gradually increase, whereas antioxidant enzyme expression and differentiation capacities gradually decrease.^71^, ^72^ Furthermore, the morphology and release of inflammatory factors are positively related to passage number, as late‐passaged MSCs are flatter and more enlarged in morphology and release higher levels of inflammatory factors, including interferon gamma (IFN*γ*) and TNF‐α.^73^, ^74^ The apoptosis rate of MSCs may also be increased via the release of nuclear factor erythroid2‐related factor 2 (Nrf2), nuclear factor (NF)‐κB, Toll‐like receptor 4 (TLR4) and other inflammatory factors.^75^ Jeong et al^76^ argued that expression of apoptosis‐related genes in bone marrow derived MSCs is notably increased at the early stage of passaging but not altered in subsequent passaging stages. Regarding this debate, more studies should be performed to investigate the underlying mechanisms for ROS regulation in MSCs during in vitro culture. H_2_O_2_, an important factor that leads to oxidative stress‐induced premature senescence (OSIPS), has been demonstrated to inhibit MSC proliferation in a concentration‐dependent manner.^6^, ^77^ H_2_O_2_ enhances the release of NF‐κB and *β*‐catenin as well as the motility of MSCs by enhancing degradation of collagen 5 and fibronectin^78^ and further facilitates osteogenic differentiation of MSCs via up‐regulation of NFE2L2 expression.^79^ After treatment with 200 μm H_2_O_2_ for 3 to 4 passages, wharton's Jelly derived MSCs become morphologically heterogeneous and irregularly flattened, with significantly up‐regulated expression of senescence markers, including SA‐*β*‐galactosidase, p53, p21, p16 and lysosomal *β*‐galactosidase.^80^ Moreover, treatment with 0.1 mmol/L H_2_O_2_ reduced the proliferative capacity and pluripotent markers of adipose derived MSCs at 2 to 5 passages as a consequence.^79^ At the same time, H_2_O_2_ induces cell cycle arrest and the DNA damage response by activating p53, p21 and p38 MAPK signalling and inactivating pRb signalling.^22^ Overall, MSC senescence is closely linked to ROS‐related pathways and senescence‐related gene expression, which lead to apoptosis and necrosis in MSCs.

## 5 STRATEGIES TO CONTROL ROS LEVELS FOR MSC FATES IN VITRO AND IN VIVO

### 5.1 Growth factors or extracellular matrix components for ROS regulation in MSCs

To eliminate the increased ROS levels and their corresponding side effects, strategies have been developed to enhance cellular activities in vitro and in vivo. Exposure to basic fibroblast growth factor (bFGF) significantly enhances the proliferative activity and reduces the cellular senescence and apoptosis in MSCs by ameliorating ROS‐induced oxidative stress.^81^ Platelet‐derived growth factor (PDGF)‐BB contributes to ROS generation to mild degrees and increases the proliferation and migration capacities of adipose‐derived MSCs.^82^ In addition, interleukin‐17 (IL‐17) activates Rac1 GTPase, NOX1, and the MEK‐ERK pathway, resulting in ROS generation, and consequently stimulates MSC proliferation, migration and motility.^83^ Ex‐4 preconditioning protects MSCs against H_2_O_2_‐induced apoptosis in a dose‐dependent manner,^84^ and pigment epithelium‐derived factor (PEDF) increases MSC survival rates in long‐term culture by attenuating p53 and p16 signalling.^85^ In addition, various types of extracellular matrix components including decellularized cell‐deposited extracellular matrix and cardiogel derived from cardiac fibroblast strongly promote the self‐renewal and differentiation of MSCs by up‐regulating the expression of intracellular antioxidative enzymes and maintaining low levels of ROS even under excessive H_2_O_2_ conditions in vitro.^86^, ^87^ In addition, Lai et al^88^ reconstructed a native extracellular matrix which is consisted of collagen, fibronectin, small leucine‐rich proteoglycans and major components of basement membrane to maintain the cell activities of MSCs via downregulation of ROS level in vitro.

### 5.2 Pharmaceuticals for ROS regulation in MSCs

To augment regimens for regulating ROS production in MSC‐based regenerative medicine, an increasing number of pharmaceuticals have been tested for particular and strong effects. As a strong antioxidant, NAC preserves MSC activities, including high self‐renewal, multipotency, a low apoptosis rate and strong cell adhesion capacity, by preventing activation of Wnt/*β*‐catenin signalling and excessive cellular ROS production.^60^, ^89^, ^90^, ^91^ Buthionine sulfoxide significantly increases albumin and ROS levels in hepatogenic MSCs, whereas NAC inhibits these related metabolic functions.^92^ However, NAC has also been demonstrated to inhibit adipocyte differentiation^59^ and to maintain the osteogenic differentiation capacity of MSCs by decreasing ROS levels.^54^ Moreover, a combination of NAC and L‐ascorbic acid 2‐phosphate promoted the growth of MSCs and suppressed oxidative stress‐induced cell death by enhancing mitochondrial integrity and function in vitro under H_2_O_2_‐induced oxidative stress.^93^ Preconditioning with Vitamin E eliminates H_2_O_2_‐induced injury in MSCs in vitro, and transplantation of these pretreated MSCs increases the proteoglycan content of cartilage matrix for tissue repair of osteoarthritis in vivo.^94^ In addition, metformin can be applied to rescue H_2_O_2_‐induced senescence, long‐term culture‐induced senescence and hypoxia/serum deprivation (Hy/SD)‐induced apoptosis in MSCs.^95^, ^96^, ^97^

Intriguingly, fullerol eliminates cellular ROS, effectively retarding the adipogenic differentiation capacity of MSCs while enhancing their osteogenic differentiation capacity.^98^, ^99^ Another compound, tricyclodecan‐9‐yl‐xanthogenate markedly increases the number of MSCs differentiating into neurons by increasing ROS levels and expression of antioxidative enzymes, but not by enhancing different SOD activities.^100^ Two medicines widely used clinically, fucoidan and carvedilol, protect in vitro MSCs from H_2_O_2_‐induced injury via inhibition of p38‐MAPK, JNK, caspase 3 and AKT as well as ROS‐related pathways.^101^, ^102^ Nicorandil attenuates Hy/SD‐induced apoptosis by reducing ROS generation, maintaining the stability of matrix metalloproteinases and decreasing the release of apoptosis‐related molecules.^103^ 5‐Azacytidine was also found to drive aged MSCs into a dynamic state by increasing their proliferative capacity and decreasing oxidative stress and DNA methylation.^29^ In addition, some drugs have been demonstrated to act as double‐edged swords in clinical therapies due to their ROS scavenger functions or ROS‐mediated cellular toxicity. For instance, low and high doses of trichostatin A or isothiocyanates have protective and harmful effects on oxidative stress in MSCs through opposite regulatory mechanisms of SOD2 and FOXO1.^104^, ^105^

### 5.3 Extracts from natural products for ROS regulation in MSCs

In addition to drugs used in the clinic, a large number of extracts from natural products, including herbs, nutritional factors, and hormones, have important functions in regulating ROS generation in MSCs. Capsaicin (8‐methyl‐N‐vanillyl‐trans‐6‐nonenamide) extracted from red pepper induces cell cycle arrest and inhibits early adipogenic differentiation by increasing ROS and reactive nitrogen species (RNS) production in a dose‐ and time‐dependent manner.^106^ Gastrodin, the main active component in *Gastrodia elata*, significantly enhances the proliferative and osteogenic differentiation capacities of MSCs while inhibiting their adipogenic differentiation capacity via suppression of ROS generation.^107^ Apocynin, a phenolic compound, partially reverses the ageing process and enhances the potential for osteogenesis in ageing MSCs by suppressing NOX.^108^ Both lycopene and *Cirsium setidens* pretreatments suppressed H_2_O_2_‐induced ROS generation and increased survival of MSCs by attenuating the phosphorylated p38‐MAPK, JNK, ATM and p53 signalling pathways.^109^, ^110^ Berberine, a natural isoquinoline quaternary alkaloid extracted from the Chinese herb Huanglian, significantly prevents H_2_O_2_‐induced apoptotic progression by reducing ROS production and levels of apoptosis‐related proteins, while increasing SOD activity and p‐AKT and Bcl‐2 levels.^111^ Astragalus polysaccharide, which is isolated from Astragalus membranaceus, is able to counter the reductions in proliferation and pluripotency and to prevent the increased apoptosis and senescence in MSCs induced by ferric ammonium citrate (FAC).^112^

In addition to the extracts isolated from plants described above, compounds derived from animals also have antioxidative capacities for ROS regulation. Melatonin maintains MSC survival and their osteogenic differentiation capacity and chondrogenesis in an IL‐1*β*‐induced inflammatory environment by down‐regulating MMP expression and up‐regulating that of SOD.^113^, ^114^ Preconditioning with melatonin significantly increased the expression of the antioxidant enzyme catalase and Cu/Zn SOD‐1 and the other factors including pro‐angiogenic and mitogenic factors in MSCs in vitro; moreover, melatonin pre‐treatment enhanced the activity of engrafted adipose derived MSCs and enhanced their therapeutic potency in a rat model of myocardial infarction (MI).^96^ Combination of melatonin and apoptotic adipose‐derived MSCs was superior to apoptotic adipose‐derived MSCs alone in protecting the kidneys from sepsis‐induced injury, as this group well‐maintained the kidney function via decreasing the protein expressions of inflammatory, apoptotic, fibrotic markers, ROS and oxidative stress.^115^ A panel of polypeptides, namely, menadione and 2,3‐dimethoxy‐1,4‐naphthoquinone, effectively causes ROS production and promotes the migration capacity of human MSCs via stimulation of ERK1/2 and JNK1/2 signalling.^116^ Furthermore, cholesterol promotes MSC senescence via the ROS/p53/p21^Cip1/Waf1^ signalling pathway,^117^ while pretreatment with high‐density lipoprotein significantly increases cell viability and reduces the rate of apoptosis by decreasing ROS production via the phosphatidylinositol‐4,5‐bisphosphate 3‐kinase (PI3K)/AKT pathway.^118^ Parathyroid hormone, which increases Ca^2+^ levels in mammals, also effectively enhances cell viability and endogenous insulin‐like growth factor 1 levels by inhibiting ROS‐related pathways.^119^ 17‐*β*‐Estradiol significantly protects MSCs against serum deprivation‐induced ROS overexpression and reduces lipid peroxidation, accompanied by a reduction in the Bax/Bcl‐2 ratio and caspase 3 level.^120^

As stem cell activities and differentiation capacities are important for MSC‐based therapeutic effects on repairing injury in vivo, drug treatments can also protect implanted MSCs from damage and then improve their biological activities for cell‐based treatments. The combination of bone marrow‐derived MSCs with plumbagin reduces the spinal cord water content after spinal cord injury by synergistically activating the Nrf2 pathway and regulating oxidative stress, inflammation and apoptosis in vivo.^121^ Injection of adipose‐derived MSCs and icariin markedly prevented apoptosis in transplanted MSCs and repaired erectile tissue structures in erectile dysfunction models, accompanied by increased levels of endothelial markers and smooth muscle markers in vivo.^122^

### 5.4 Gene modification for regulating ROS generation in MSCs

Gene modification is widely used in regenerative medicine for reprogramming somatic cells into pluripotent cells. Recently, this technology has also been applied to alter intracellular ROS levels to maintain stem cell properties in vitro and in vivo. Although oxidative stress can easily induce diverse nuclear malformations, overexpression of TERT in MSCs protects them from nuclear damage and significantly improves the basal activities of antioxidant enzymes, SOD and catalase in MSCs.^123^ Knockout of exon 4 of pNO40/PS1D in MSCs results in elevated ROS levels and p16 and Rb expression, consequently accelerating ageing and osteogenic differentiation defects.^124^ Similarly, MSCs isolated from Tks4 knock‐out mouse have reduced osteogenic and adipogenic differentiation capacities compared with those isolated from wild‐type mice.^125^ Although knockdown of NOX4 suppressed ROS production and adipogenic differentiation in MSCs,^126^ Kim et al^43^ demonstrated that NOX4 silencing did not inhibit MSC adipocyte differentiation. Modification of genes in MSCs not only exerts important effects on their differentiation capacities but also influences cell senescence or apoptosis. Overexpression of TERT also protects long‐term cultured MSCs from senescence via similar mechanisms.^127^ Overexpression of PPARγ co‐activator 1α increases the survival rate of MSCs by reducing intracellular and mitochondrial ROS, even under conditions of high glucose, hypoxia and serum deprivation.^128^ Similarly, overexpression of proteasome subunit beta 5 (PSMB5) maintains the differentiation capacity of late‐passage MSCs, even under exposure to H_2_O_2_,^129^ and overexpression of heme oxygenase 1 in MSCs significantly attenuates H_2_O_2_‐induced injury of retinal ganglion cells by decreasing levels of cellular ROS and proapoptotic proteins. Thus, modified MSCs are highly effective at attenuating retinal I/R injury by maintaining structural thickness and decreasing apoptosis.^130^ In addition to general virus‐mediated overexpression of critical genes, microRNAs (miRNAs) and small molecules contribute to the regulation of MSC activities by modulating ROS levels. Overexpression of microRNA‐34a, a p53‐targeted miRNA, significantly enhances apoptosis, impairs cell vitality and aggravates senescence by promoting mitochondrial dysfunction and activating intrinsic apoptosis pathways; unfortunately, the harmful effects on MSCs can only be partially abolished by NAC.^131^ Specific miR‐210 inducers activate ERK1/2 and AKT pathways in MSCs but inhibit the c‐Met pathway, thus eliminating ROS‐induced apoptosis in MSCs.^82^, ^132^ Small inhibitory RNAs (siRNAs) targeting insulin‐like growth factor 1 and Nrf‐2 counteract the protective effects of parathyroid hormone in MSCs in vitro by enhancing ROS generation.^119^ In contrast, siRNA targeting caspase 3 strongly attenuates H_2_O_2_‐induced apoptosis of MSCs,^133^ and siRNA targeting p16 reverses LPS‐induced DNA damage and MSC senescence.^134^ As let‐7b has been identified as one of the mediators targeting caspase‐3 for eliminating ROS‐induced apoptosis and autophagy in MSCs, administration of let‐7b‐modified MSCs maintained ventricular function and accelerated myocardial repair in rat cardiac IR model.^135^ Intra‐myocardial injection of adenovirus‐ecSOD transfected MSCs exerted cardiac protection against MI partly via reduction of oxidative stress and enhancing the survival rate of MSCs.^136^

## 6 CONCLUSIONS

**Figure 2:**
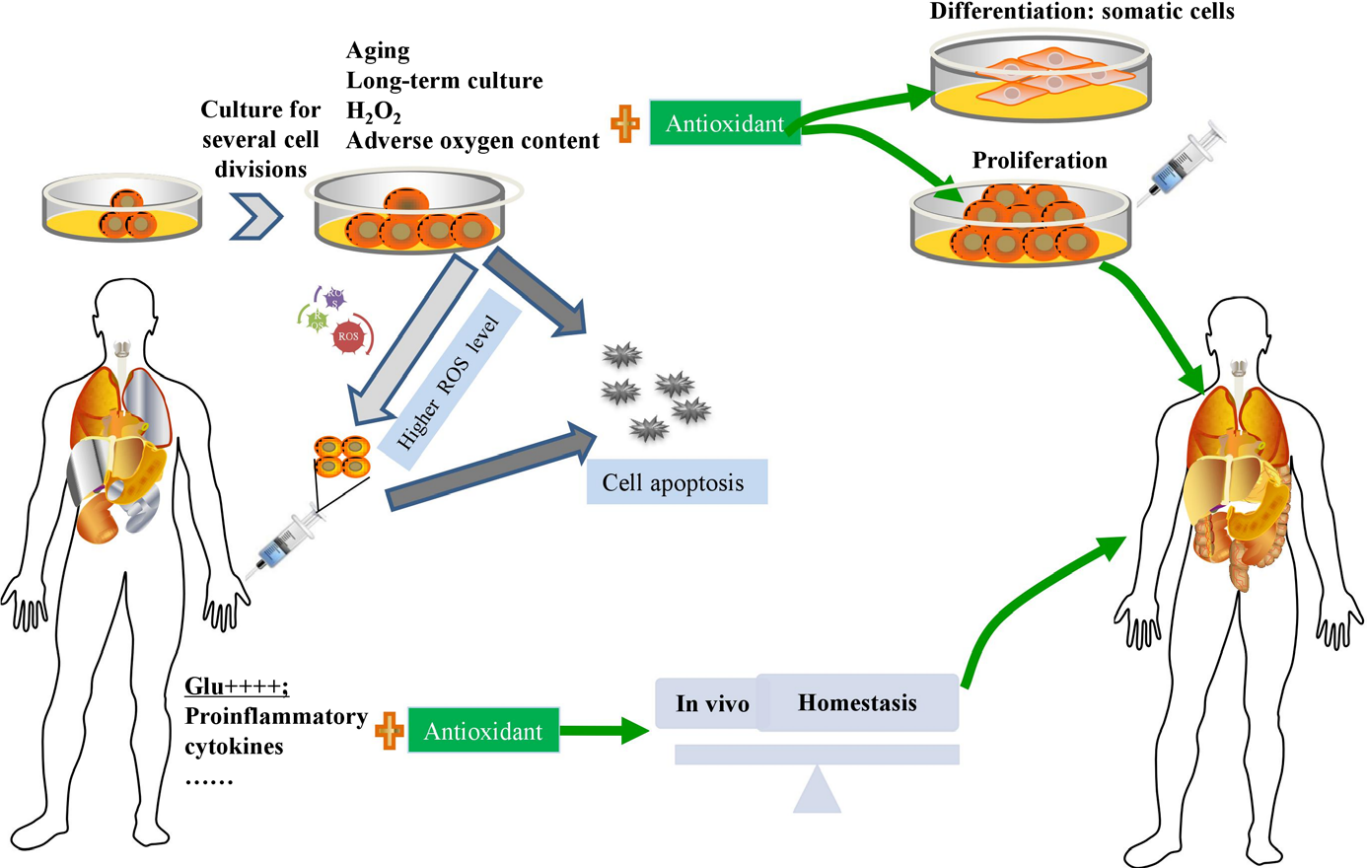
Antioxidants for MSCs contribute to the regenerative applications of MSCs

Although MSCs serve as promising resources for repairing tissue or organ functions, excessive ROS can be generated during MSC expansion and differentiation processes. In addition, MSC properties are partially dependent on oxidative stress; hence, novel approaches for optimizing ROS generation in MSCs can contribute to their regenerative applications (Figure 2). Multiple antioxidative enzymes and non‐enzymes participate in the physiological regulation of the redox balance of MSCs, and high levels of intracellular or extracellular ROS may trigger the DNA damage response, senescence and loss of potency of MSCs in vitro. These effects are strongly prohibitive for MSC proliferation and differentiation and thus not beneficial for MSC transplantation in vivo for experimental or clinical therapy. Moreover, transplanted MSCs face harsh environments that induce apoptosis and impair migration, markedly limiting the efficacy of MSC‐based therapeutics, thus the improvement of survival and antioxidant ability of MSCs in vivo should be confirmedMost importantly, clarification of the regulatory mechanisms maintaining the MSC redox balance in vitro and in vivo will help to retain stem cell activities even under adverse conditions. Moreover, improving the culture conditions for various resources derived from MSCs is critical for maintaining pluripotency and enhancing their differentiation capacity in vitro. Although the effects of some antioxidants vary somewhat among different stem cell types or different culture conditions, a correlation between the antioxidant defence level and stem cell fate decision does exist. Fortunately, some natural extracts that rarely exert side‐effects can be applied as strong antioxidants in oxidative stress‐induced microenvironments. In addition, gene modifications that were originally used to generate stem cells from somatic cells also serve as advanced technologies for ROS regulation in MSC‐based tissue engineering. All these strategies potentially help to improve the cell activities of MSCs but decrease the ROS level in vivo for enhancing the therapeutic effects. On the other hand, it is still necessary to further elucidate the mechanisms of physiological and pathological ROS generation, external ROS regulation via of multiple compounds and specific gene modifications. Only in this way will we effectively regulate ROS levels to take advantage of the mass multiplication and strong differentiation capacities of MSCs, which will allow for harnessing the immortality of MSCs for transplantation and therapy in regenerative medicine.

## CONFLICT OF INTEREST

The authors declare no competing financial interests.
